# Tax Benefits for Preventing and Supporting Catastrophic Health Expenditures: Ex-Ante versus Ex-Post Governmental Financial Support

**Published:** 2019-08

**Authors:** Sung Man YOON, Byung Wook JUN

**Affiliations:** 1. Department of Business Administration, Seoul National University of Science and Technology, Seoul, South Korea; 2. Graduate School of Science in Taxation, University of Seoul, Seoul, South Korea

## Dear Editor-in-Chief

In Korea, non-payment healthcare expenditures (NPHEs) not covered by national health insurance are very high (amounting to about 40% of all medical expenses). This is caused by the continued expansion of NPHEs by private providers in the absence of an NPHE management system (1).

The 2014 average of the member countries of the Organization for Economic Co-operation and Development (OECD) for the ratio of public funds to household healthcare expenditures (HEs), an indicator of healthcare insurance (HI) guarantee levels, was 72.6%, while Korea’s average was just 56.5% (2). As a result, Korea’s private HI market is more active than that of other OECD members, and the Korean government continues to provide tax incentives to induce households to take out private HI for excessive healthcare expenses.

Therefore, the purpose of this study was to analyze the effectiveness of the governmental financial support system to prevent catastrophic health expenditures (CHEs) in households. Prior studies on CHEs mostly focused on socioeconomic variables or demographic characteristics. However, this study aims to focus on HI finance in terms of taxes.

The government’s tax support for CHEs can be largely divided into proactive (ex-ante) and reactive (ex-post) support at the time of governmental supports. Ex-ante tax support encourages people to take out private HI if they cannot cover their HEs with their household income, such as in the case of CHEs. On the other hand, ex-post tax provides support for HEs in the form of tax relief when an individual’s HEs amount to more than 3% of his or her income and his or her expenditures are greater than a certain amount. Therefore, household HEs are supported only when they are not an unconditional tax support but when they are at an unbearable level compared to the income level of households.

CHE standards are still different for scholars, and some argue that the government should financially support households with annual HEs of 10% or more of the household income (3–5). Some others argue that HEs should not exceed 20% of the household income (5). As our analysis results, Table 1 defines the scope of CHEs as >10%, 20%, 30%, and 40% and shows the results of an effectiveness analysis for EX_Ante_Support and EX_Post_Support. EX_Ante_Support represents negative coefficients at a statistically significant level in all columns. The coefficients represent −0.045±0.007 (*P*<0.001), −0.049±0.013 (*P*<0.001), −0.053±0.019 (*P*<0.001), and −0.059±0.026 (*P*<0.05), for CHEs>10%, 20%, 30%, and 40%, respectively. This means that a system that supports a certain portion of the insurance policy in the form of tax cuts reduces the likelihood of CHE outbreaks in case of disease or accidents.

**Table 1:** Results of random-effect ordered logistic regression

***Variables***	***Dependent variable: CHE = Occurrence of CHE [0, 1]***			
	***CHE > 10%***	***CHE > 20%***	***CHE > 30%***	***CHE > 40%***
Intercept	−7.618(0.561)^***^	−10.135(0.802)^***^	−12.878(0.128)^***^	−1.470(1.215)^***^
EX_Ante_Support	−0.045(0.007)^***^	−0.049(0.013)^***^	−0.053(0.019)^***^	−0.059(0.026)^**^
EX_Post_Support	0.032(0.002)^***^	0.030(0.003)^***^	0.026(0.005)^***^	0.016(0.008)^**^
STD(Healthcare_Exp)	0.324(0.027)^***^	0.319(0.039)^***^	0.319(0.048)^***^	0.322(0.058)^***^
Med_Benefit_Recipient	−0.501(0.179)^***^	−0.648(0.250)^***^	−0.921(0.303)^***^	−1.135(0.375)^***^
Log(Asset)	0.021(0.012)^*^	0.020(0.017)	0.021(0.020)	0.050(0.025)^**^
Log(Income)	−1.692(0.073)^***^	−2.170(0.106)^***^	−2.614(0.138)^***^	−2.977(0.170)^***^
Family_Size	0.165(0.037)^**^	0.188(0.055)^***^	0.335(0.070)^***^	0.419(0.084)^***^
House_Holder [0,1]	0.166(0.084)^**^	0.185(0.124)	0.074(0.154)	0.108(0.184)
Metropolitan [0,1]	0.345(0.077)^***^	0.433(0.114)^***^	0.278(0.142)^**^	0.341(0.170)^**^
Married [0, 1]	0.051(0.087)	0.179(0.130)	0.030(0.163)	0.095(0.198)
Age	0.029(0.003)^***^	0.023(0.005)^***^	0.017(0.006)^***^	0.006(0.007)
Education	−0.205(0.090)^**^	−0.177(0.139)	−0.033(0.175)	−0.080(0.213)
Female [0, 1]	−0.208(0.107)^*^	−0.368(0.153)^**^	−0.466(0.185)^**^	−0.646(0.224)^***^
2014Dummy [0, 1]	0.277(0.069)^***^	0.432(0.101)^***^	0.529(0.128)^***^	0.440(0.153)^***^
Log Likelihood	−3888.59	−2071.82	−1347.16	−977.81
Observations	13,496			

Note:

1) ( ) is standard error and *** <0.001, ** <0.05, * <0.1 (two-tailed)

2) Healthcare expenditure in CHE includes oriental medicine, cosmetic surgery, dental treatment, hospitalization, outpatient care, medications, and health examination expenses

Thus, the effectiveness of the Government’s proactive CHE support system exists. In particular, when the CHE threshold increases from 10% to 40%, the coefficients also increase, suggesting that the lower the household income level, the greater the effect of such proactive support.

In addition, as a reactive support scheme, *EX_Post_Support* represents a positive coefficient at a statistically significant level in all columns. For CHEs>10%, 20%, 30%, and 40%, the coefficient represents 0.032±0.002 (*P*<0.001), 0.030±0.003 (*P*<0.001), 0.026±0.005 (*P*<0.05), and 0.016±0.008 (*P*<0.05), respectively. This means that an increase in HE results in an increase in tax benefits that are provided in the form of a proportionate tax reduction.

Comparing the absolute value coefficient of *EX_Ante_Support* to that of *EX_Post_Support* reveals that *EX_Ante_Support* is relatively more effective, suggesting that proactive support may be effective in preventing or supporting CHEs.

The above analysis indicates that the government’s proactive support system for CHEs is effective in preventing such expenditures and that; after all, it provides tax reduction benefits under CHEs. In particular, the lower the income level (*Log(Income)*) and the greater the number of family members (*Family_Size*), the more likely it is the CHEs will occur, presenting a result that corresponds to prior studies on CHEs. These findings mean that efficient resource allocation should be made through government tax expenditures for national health insurance as a precondition to prevent CHEs, especially for people with a larger number of family members and lower income levels.

## References

[1] Lee HO (2018). Effect of Four Major Severe Diseases Benefit Expansion Policies on the Health Care Utilization and Catastrophic Health Expenditure. Korean J Soc Welf, 70(1): 89–116. (In Korean)

[2] OECD health data. http://stats.oecd.org/

[3] Berki SE (1986). A look at catastrophic medical expenses and the poor. Health Aff (Millwood), 5(4): 138–145. 3102333 10.1377/hlthaff.5.4.138

[4] Feder J Hadley J Holahan J (1981). Insuring the Nation’s Health: Market Competition, Catastrophic and Comprehensive Approaches. Washington, DC: Urban Institute Press.

[5] Feldstein MS (1971). A new approach to national health insurance. J Public Interest, 23(Spring): 93.

